# Comparative Analysis of Neurodegeneration and Axonal Dysfunction Biomarkers in the Cerebrospinal Fluid of Patients with Multiple Sclerosis

**DOI:** 10.3390/jcm11144122

**Published:** 2022-07-15

**Authors:** Agnieszka Kulczyńska-Przybik, Maciej Dulewicz, Julia Doroszkiewicz, Renata Borawska, Ala Litman-Zawadzka, Daria Arslan, Alina Kułakowska, Jan Kochanowicz, Barbara Mroczko

**Affiliations:** 1 Department of Neurodegeneration Diagnostics, Medical University of Bialystok, 15-269 Bialystok, Poland; maciej.dulewicz@umb.edu.pl (M.D.); julia.doroszkiewicz@umb.edu.pl (J.D.); renata.borawska@umb.edu.pl (R.B.); ala.litman-zawadzka@umb.edu.pl (A.L.-Z.); arslan.daria@gmail.com (D.A.); mroczko@umb.edu.pl (B.M.); 2 Department of Neurology, Medical University of Bialystok, 15-269 Bialystok, Poland; alina.kulakowska@umb.edu.pl (A.K.); jan.kochanowicz@umb.edu.pl (J.K.); 3 Department of Biochemical Diagnostics, Medical University of Bialystok, 15-269 Bialystok, Poland

**Keywords:** neurodegeneration, multiple sclerosis, neurofilament light chain, reticulon 4, tau protein

## Abstract

Background: Given the significant role of neurodegeneration in the progression of multiple sclerosis (MS) and insufficient therapies, there is an urgent need to better understand this pathology and to find new biomarkers that could provide important insight into the biological mechanisms of the disease. Thus, the present study aimed to compare different neurodegeneration and axonal dysfunction biomarkers in MS and verify their potential clinical usefulness. Methods: A total of 59 patients, who underwent CSF analysis during their diagnostics, were enrolled in the study. Quantitative analysis of neurodegeneration biomarkers was performed through immunological tests. Oligoclonal bands were detected by isoelectric focusing on agarose gel, whereas the concentrations of immunoglobulins and albumin were measured using nephelometry. Results: Our studies showed that NfL, RTN4, and tau protein enabled the differentiation of MS patients from the control group. Additionally, the baseline CSF NfL levels positively correlated with the tau and MRI results, whereas the RTN4 concentrations were associated with the immunoglobulin quotients. The AUC for NfL was the highest among the tested proteins, although the DeLong test of the ROC curves showed no significant difference between the AUCs for NfL and RTN4. Conclusion: The CSF NfL, RTN-4, and tau levels at the time of diagnosis could be potential diagnostic markers of multiple sclerosis, although NfL seems to have the best clinical value.

## 1. Introduction

Multiple sclerosis (MS) is a chronic neurological disease characterized by focal inflammatory lesions in the gray and white matter, as well as progressive, diffuse neurodegeneration in the entire brain. The evidence from preclinical studies has shown that neurodegeneration is already apparent in MRI examination at the time of diagnosis [1]. Moreover, it is suggested that early neurodegeneration developing from disease onset is typical for patients with a primary progressive form of MS, whereas, in patients with secondary progressive disease, the gradual process of neurodegeneration develops after many years (15–25 years) [2]. The mechanisms of concomitant neurodegeneration are still not sufficiently understood. It is believed that inflammation can drive neurodegeneration at any stage of the disease [3]. On the other hand, neurodegeneration in MS may arise independently of inflammation and may even be the primary cause of CNS damage in this disorder [4]. Importantly, early and progressive loss of axons is related to the irreversible neurological disability in MS patients. Therefore, a better understanding of the neurodegenerative pathways and mechanisms that promote neurodegeneration in MS appears to be highly important. Most of the studies related to biomarkers in MS focus on proteins reflecting an inflammatory state. We would like to emphasize that searching for and examining indicators of neurodegeneration are equally essential in this disease. Identifying objective and more sensitive biomarkers is necessary for screening, for the development of new individualized therapies, for early diagnosis, and for accurate disease prognosis. The application of markers in clinical practice has significantly increased in the last few decades [5].

**Figure 1:**
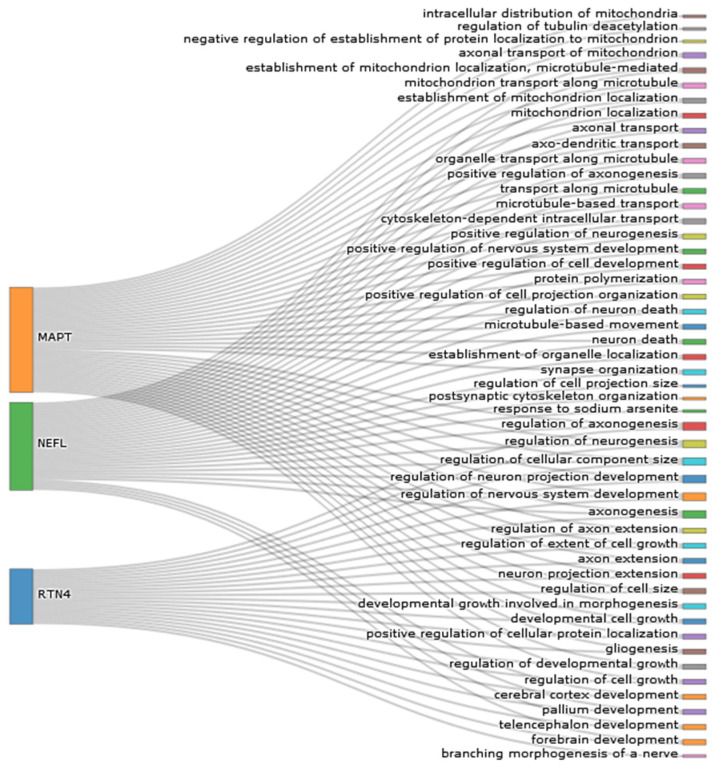
The shared functions of the tau (MAPT), NfL (NEFL), and RTN4 proteins.

One neurodegeneration hallmark in MS patients is axonal damage and loss, which leads to brain and cervical atrophy, cortical thickening, disability, and cognitive dysfunction [6,7,8,9]. A growing body of evidence has shown that neurofilament light chain (NfL) is a robust marker reflecting the extent of neuroaxonal damage [6,7]. NfL has been identified as a biomarker of disease activity in MS [8,9,10]. It has been demonstrated that an elevated concentration of NfL is correlated with brain atrophy and loss of spinal cord volume, prediction of the future course of the disease, and response to treatment [9,11]. However, this marker is not specific to MS and can be detected in many other neurodegenerative diseases. Moreover, in the literature, other biomarkers of neurodegeneration have also been proposed; therefore, it seems to be interesting to compare them in MS. We chose three molecules on the basis of the literature review and an analysis of the functional proteins reflecting neurodegenerative pathology (Figure 1). With these aspects in mind, the purpose of the present study was a comparative analysis of different neurodegeneration biomarkers in patients with MS and an assessment of the potential diagnostic utility of these markers. To the best of our knowledge, this is one of the first studies to compare biomarkers of neurodegeneration and axonal dysfunction, such as NFL, RTN4, and tau, in multiple sclerosis.

## 2. Materials and Methods

### 2.1. Study Population—Clinical Assessment

The Ethics Committee of Bialystok University approved the study (No. R-I-002/103/2019), and all the patients signed an informed consent form before any procedure. The study population consisted of 59 subjects with neurological disorders (36 women and 23 men; median age of 36 years), including patients with MS (*n* = 37) and subjects with other noninflammatory neurological diseases as a control group (*n* = 22) (Table 1). Study participants were diagnosed and treated in the Department of Neurology Medical University of Bialystok, Poland. Patients underwent clinical evaluations, neurological examinations, neuroimaging tests (MRI - magnetic resonance imaging), and routine blood and CSF screening tests. Paired CSF and serum samples from the MS patients were collected between 2018 and 2020. All patients in the study were enrolled at the time of the diagnosis.

In this investigation, the diagnosis of MS was based on the MacDonald criteria (2017) [12]. The subjects met the following criteria to be enrolled in the study: neurological stability with no evidence of relapse or corticosteroid treatment within 30 days prior to collecting the biological material; age between 18 and 65 years old; newly diagnosed and treatment-naïve at the time of the diagnosis; definite relapsing–remitting MS diagnosed by a neurologist. Individuals were excluded from enrollment if the following criteria were present: diagnosed active systemic infectious diseases or other coexisting autoimmune neurodegenerative diseases; treatment with vitamin and/or antioxidant supplements (which could alter the laboratory biomarkers evaluated); history or presence of malignancy of the central nervous system; incomplete medical history or radiological data.

All MS patients included in the study were diagnosed with relapsing–remitting MS. They had a history of at least one clinical attack, and there was no evidence of dissemination in time or atrophy according to magnetic resonance imaging (MRI). A radiologist examined all the MRI scan data of the enrolled patients. Patients were followed up clinically and radiologically according to routine clinical practice. The presence of oligoclonal bands (OCBs) in the cerebrospinal fluid and serum was also assessed. The disease severity in patients with MS was assessed using the Expanded Disability Status Scale (EDSS). All evaluations were rated between 1 and 2 points, indicating an early stage of the disease. Since the CSF was collected during the diagnostic process, none of the patients were on disease-modifying therapy during lumbar puncture. Matched CSF and serum samples from the patients were collected only once during disease diagnosis. Moreover, 23 out of 37 patients had OCBs in the CSF but not in serum (pattern type 2), whereas five had OCBs in the CSF and serum, with additional OCBs in the CSF (pattern type 3).

The control group consisting of 22 neurological patients (11 females and 11 males) was carefully selected on the basis of neurological, neuropsychological, and laboratory (blood and CSF) examinations, which allowed excluding the organic background of symptoms. Additionally, the concentrations of albumin and immunoglobulins in the CSF and serum were simultaneously assessed to calculate their quotients. In the control group, 16 patients had no bands in the CSF and serum (pattern type 1), whereas five had the same pattern of OCBs in the CSF and serum (pattern type 4).

### 2.2. Biomarker Analysis

CSF samples were drawn by lumbar puncture (LP), performed in the L3/L4 or L4/L5 interspace at the time of diagnosis. CSF was collected in sterile polypropylene tubes, centrifuged for 10 min at 2000× *g*, divided into 0.5 mL aliquots, and immediately frozen at −80 °C until analysis. After lumbar puncture, patient demographic and clinical data were stored. The tested biomarkers measured in the current study were assessed using the same batch of reagents in the Department of Neurodegeneration Diagnostics, Medical University of Białystok, Poland. CSF samples were run in duplicate. The quantitative analysis of tested proteins was prepared following each protein manufacturer’s instructions. CSF RTN-4 was measured using a human RTN4 (Reticulon 4) ELISA kit (Wuhan, Hubei, China), CSF NfL was analyzed using a sandwich ELISA NF-light UmanDiagnostic kit (Umeå, Sweden), and CSF tau was determined using an Innotest Fujirebio kit (Europe, Gent, Belgium) following the manufacturer’s instructions.

CSF and serum concentrations of albumin and immunoglobulins were measured using a nephelometer (Optilite; The Binding Site). Albumin and immunoglobulin quotients were calculated (Q_Alb_, Q_IgG_, Q_IgA_, and Q_IgM_) to assess the integrity of the blood–CSF barrier and intrathecal immunoglobulin production. Furthermore, the oligoclonal bands were assessed using isoelectric focusing on agarose gel (Hydragel 3 CSF Isofocusing; Hydrasys; Sebia) by immunoblotting, following the manufacturer’s protocol. Patients were classified as OCB-positive if they had more than one CSF OCB, but considered OCB-negative if they had 0–1 CSF OCBs.

### 2.3. Statistical Analysis

Statistical analysis was conducted using the PMCMRplus package in the statistical software R [13]. A *p*-value below 0.05 was considered statistically significant. Furthermore, Figure 1 was created in R Studio using packages: clusterProfiler [14] and Plotly [15]. After obtaining data about GO enrichment analysis for biological processes, functions typical for the tested biomarkers were visualized. The chi-square, Shapiro–Wilk, and Levene tests were employed to verify the normality of the distribution. Since the CSF levels of NfL and immunoglobulins, as well as immunoglobulin quotients, were not normally distributed, they were transformed before further analysis. The study groups were compared with regard to age, gender distribution, and CSF levels of RTN-4, NfL, and tau. Student’s *t*-test was used to compare the variables between the MS and control groups. The results are reported as the mean ± SD depending on the normality distribution. A Pearson correlation analysis was performed to examine whether the relationships between markers of MS pathology (OCBs, immunoglobulin quotients, and demyelination in MRI) and neurodegeneration biomarkers were different in patients with MS and controls. Additionally, receiver operating characteristic (ROC) curve analysis was performed to determine the diagnostic usefulness of the tested proteins as potential biomarkers in MS. DeLong’s test was used to determine whether the AUCs of tested biomarkers were statistically significantly different.

## 3. Results

### 3.1. Characteristics of Participants

The characteristics of participants included in our investigation are reported in Table 1. In the whole study group, patients with the relapsing–remitting form of MS were younger and were more likely to be female than controls. The control group tended to be older and had a more equal proportion of males and females compared to the MS group. Isoelectric focusing in 28 out of 37 patients with MS revealed type 2 and type 3 oligoclonal bands, while the remainder of the group was negative. Moreover, T2 and FLAIR demyelination lesions were found in MRI scans in the majority of patients with MS as compared to controls. An active inflammatory process was excluded on the basis of biochemical tests (e.g., CRP, WBC, albumin, and immunoglobulins). Moreover, the presence of comorbidities at the time of the diagnosis was assessed. In MS patients, the following comorbidities were found: hypertension, spine disorders, thyroid disorders, type 2 diabetes, and eye diseases (Table 1).

### 3.2. Quantification of CSF Neurodegeneration Biomarkers

**Figure 2:**
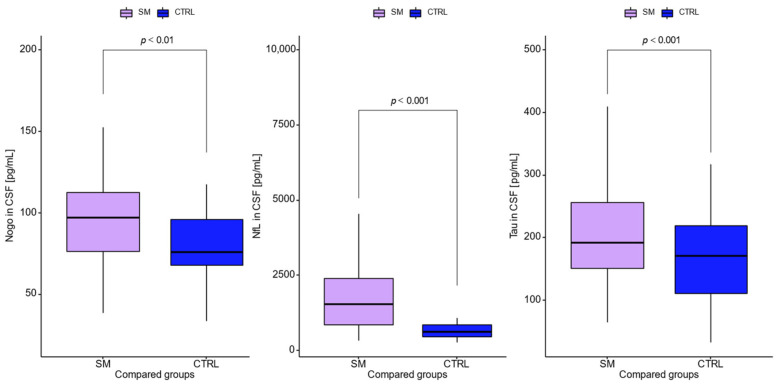
CSF concentrations of RTN4, NfL, and tau in MS patients and control group.

Table 2 presents the levels of CSF albumin and immunoglobulins, as well as their quotients, and the CSF concentrations of neurodegeneration biomarkers. The CSF concentrations of IgG (*p* < 0.001) and IgM (*p* = 0.002), as well as Q_IgG_ (*p* < 0.001), Q_IgM_ (*p* < 0.001), and Q_IgA_ (*p* = 0.05), were significantly higher in relapsing–remitting MS as compared with controls. A comparison of the CSF levels of tested biomarkers is presented in Figure 2. The CSF concentration of RTN4 was significantly higher in the group with multiple sclerosis than in the controls. Similarly, other CSF biomarkers reflecting neurodegenerative changes (NfL and tau) were significantly elevated in MS patients as compared to controls. However, among all tested biomarkers, the highest difference between MS patients and controls was observed for NfL (*p* < 0.001). Moreover, the ratios of NfL to RTN4 and tau were calculated. Only the NFL/RTN4 ratio was significantly different between the MS and control groups (*p* = 0.03).

### 3.3. Relationships between Tested Neurodegeneration Biomarkers and Immunological Parameters in Patients with MS

**Figure 3:**
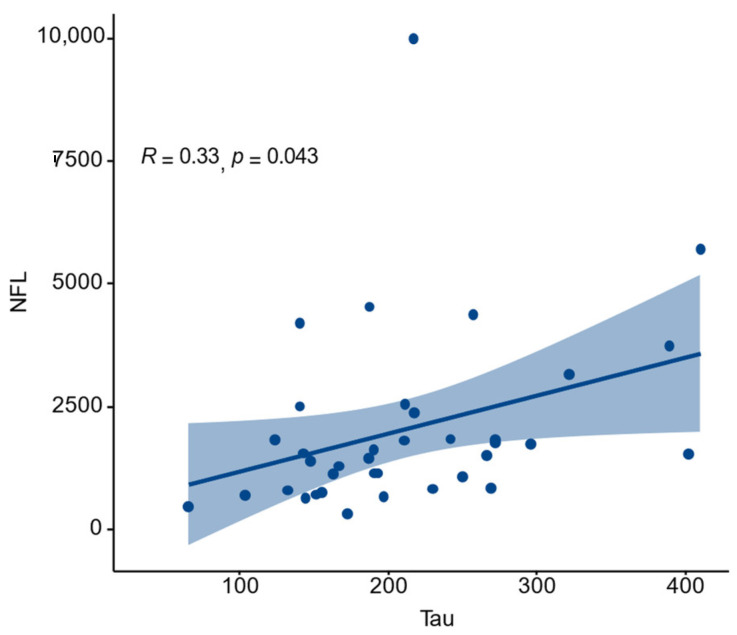
The correlation between NfL and tau protein in patients with MS.

In the group of all the MS patients, significant correlations between log NfL and tau (*r* = 0.45; *p* = 0.01) (Figure 3) and demyelinating changes assessed by MRI (*r* = 0.41; *p* = 0.01) were observed. Additionally, the CSF RTN4 level was positively associated with log Q_IgA_ (*r* = 0.51; *p* = 0.004) and log Q_IgM_ (*r* = 0.43; *p* = 0.02). There were no correlations with other variables such as age, sex, and oligoclonal bands.

### 3.4. Diagnostic Usefulness of Tested Neurodegeneration Biomarkers in Multiple Sclerosis

Table 3 presents the results of the receiver operating characteristic curve (ROC) analysis, with areas under the ROC curves (AUC) and predictive values for the tested CSF biomarkers. The results showed that CSF NfL had a higher diagnostic value in MS patients, with an area under the ROC curve (AUC) of 0.836 (0.544–0.824; *p* < 0.001), sensitivity of 0.78, and specificity and 0.77, compared to CSF RTN4 and tau. AUC values for the CSF levels of RTN4 and tau were comparable (AUC = 0.684, 95% CI: 0.609–0.878, *p* = 0.005 vs. AUC = 0.630, 95% CI: 0.480–0.782, *p* = 0.046, respectively).

**Figure 4:**
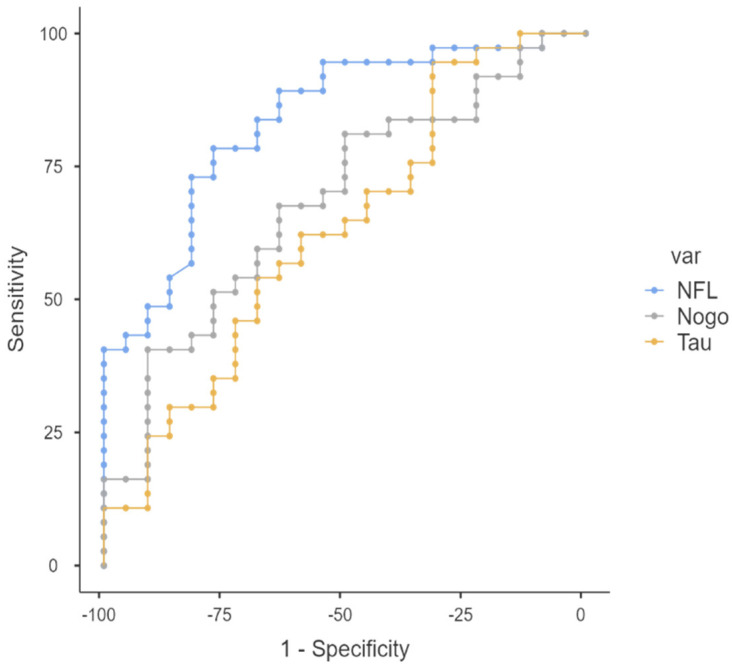
Diagnostic value of CSF neurodegeneration biomarkers in MS patients.

The analysis of differences between AUCs according to DeLong’s test in the MS and control groups showed a significant difference between NfL and tau (AUC difference = 0.206, 95% CI: 0.055–0.357, *p* = 0.008). Furthermore, the DeLong test of the ROC curves showed no significant difference between the AUCs for RTN4 and tau (AUC difference = 0.054, 95% CI: 0.100–0.208, *p* = 0.491) or the AUCs for NfL and RTN4 (AUC difference = 0.152, 95% CI: 0.008–0.312, *p* = 0.063) despite the highest value of the area under the ROC curve for NfL (Figure 4).

## 4. Discussion

Mounting evidence indicates that neurodegeneration is also a serious problem and an immense challenge in MS. Clinical observations and experimental studies have revealed that this process begins at disease onset, expands with disease progression, and exists independently of the MS type [3]. Thus, reliable biomarkers reflecting this pathology seem to be important for diagnosing and monitoring the course of MS. The search for biomarkers in MS is a very active field of research. In the available literature, many proteomic indicators of neurodegeneration are described; however, there remains a lack of biomarkers allowing for more accurate diagnosis. Thus, we examined select neurodegenerative biomarkers in MS and compared their clinical usefulness. Our study revealed significantly elevated concentrations of all tested biomarkers reflecting neurodegeneration in the MS group, among which NfL seemed to have the highest diagnostic value as a biomarker at disease onset. The diagnostic power of RTN4 and tau as clinical tests was seemingly comparable and lower than that of NfL. A relationship of elevated concentrations of NfL and tau with MRI demyelination lesions was demonstrated. Moreover, the study showed an association between the levels of RTN4 and immunoglobulin quotients, reflecting a more accelerated immunological response. Given the abovementioned correlations and the functions of both proteins (NfL and RTN4), a combined analysis of both biomarkers potentially allows for improving the diagnosis of MS, although our study did not confirm this hypothesis.

In our study, we compared a novel, potential neurodegenerative biomarker with well-investigated markers to verify their application in supporting the diagnosis of MS. Our study revealed increased NfL, RTN4, and tau protein concentrations in MS patients compared to controls. These findings are in line with other studies [16,17,18]. Interestingly, we noticed that NfL showed the highest ability to differentiate between the studied groups among the three examined neurodegeneration biomarkers. Our findings revealed that NfL was the earliest and most sensitive biomarker of neurodegeneration at disease onset compared with the other tested biomarkers. However, higher CSF levels of all tested biomarkers at MS diagnosis may be useful for predicting disease prognosis. NfL is an abundant cytoskeletal protein expressed by central and peripheral neurons [10,19]. As a consequence of axonal injury, larger amounts of NfL are released into the cerebrospinal fluid [20], with approximately 40-fold lower amounts released into the blood [21]. Moreover, the biomarker concentration changes over the course of the disease, increasing as the disease worsens and decreasing with improvement. Several studies have demonstrated increased NfL levels during MS relapse, disability, and disease progression [7,22,23]. Bjornevik et al., in a group of asymptomatic patients with increased baseline and pre-symptomatic blood NfL levels, demonstrated a higher risk of developing MS [24]. Hakansson et al. compared the predictive value of CSF NfL levels and different molecules in MS progression, and they reported that NfL may be an independent predictor of conversion from clinically isolated syndrome (CIS) to MS [25]. In line with these results, another group of researchers demonstrated that NfL concentrations above 500 pg/mL allow for predicting the conversion from isolated clinical events to MS [26]. A similar observation was reported for tau protein. Baseline CSF tau concentrations have predictive value along with MRI results at the time of diagnosis [27]. Interestingly, data from clinical studies revealed that disease-modifying therapies may influence the NfL concentration [28,29]. In the treated group of patients, NfL concentration decreased, highlighting it as a valuable treatment response biomarker in clinical trials [30]. Similarly, longitudinal changes in serum NfL levels indicate its usefulness as a marker in making therapeutic decisions [31].

Other studies have also demonstrated a correlation between NfL levels and radiological findings, in agreement with our findings. We observed a significant association between CSF NfL levels and MRI demyelinating lesions in treatment-naïve RRMS patients, which may indicate a worse prognosis in this group of patients. A similar correlation was not observed for tau and RTN4 levels. A relationship of elevated levels of NfL with brain atrophy and spinal cord volume loss has been described in the literature [9,22,32,33]. The studies of Chitnis et al. showed a significant correlation of early NfL levels with 10-year MRI results, including T2-weighted lesion volume and atrophy. The authors postulated that the relationship between baseline levels of NfL and long-term outcomes of MRI might allow for the stratification of patients at higher risk of more severe disease and needing more aggressive treatment [33]. Similar conclusions were reached in a multicenter longitudinal study carried out on 814 patients with RRMS and CIS, where serum NfL levels correlated with the number of T2 and Gd^+^ lesions at baseline and future clinical relapses. Despite NfL reflecting the axonal injury leading to brain volume loss and playing a pivotal role in the development of patient disability, a relationship with the Expanded Disability Status Scale (EDSS) was not found.

Unexpectedly, even though RTN4 plays a role in two major pathomechanisms underlying MS (i.e., axonal degeneration and demyelination), it seemingly has less diagnostic significance in the disease than NfL, particularly at disease onset. Considering the in vivo studies of Nikic et al. [34], which demonstrated that axonal degeneration can precede demyelination and that RTN4 is an abundant component of myelin sheaths in the central nervous system (CNS), which take part in myelination [35], we can speculate that the concentration of RTN4 could increase in a more advanced stage of disease when there are more demyelinating changes. Moreover, previous studies revealed that RTN4 seems to be an important modulator of neuroinflammation in microglia/macrophages [36] and can inhibit neurite outgrowth and prevent the regeneration of severed axons [37]; both processes are also more potentiated in the later phase of disease, which may partially explain the lower accuracy of this biomarker in newly diagnosed patients with MS. Our results are in accordance with other studies, which showed elevated RTN4 levels in the chronic active demyelinating lesions of brain tissues, cerebrospinal fluid, and blood if patients with multiple sclerosis [16,17,38,39]. Evidence supporting the application of this protein as a biomarker in MS was also provided by studies reporting the presence of RTN4 in CSF of patients regardless of stage and type of disease (remitting–relapsing and primary progressive MS). This protein was found in the early phase of the disease and in a long-lasting, advanced stage of MS [16], indicating the possibility of using RTN4 to monitor the course of the disease. However, contrasting data are also available [40]. In light of available findings, it appears crucial to examine the concentration of RTN4 as a predictive marker reflecting the therapeutic effect. Studies on the MS mouse model EAE revealed that this protein could also be a potential therapeutic target, as blocking Nogo-A receptors ameliorated the symptoms of the disease, enhanced functional recovery, and increased axonal sprouting, as well as remyelination [41,42].

We found a positive correlation between immunoglobulins quotients and RTN4A concentrations, supporting the theory that RTN4A is more strongly involved in the central nervous system (CNS) inflammatory state and increases due to the potentiated immunological response after demyelination. Abundant overexpression of receptor NgR1 was demonstrated in the microglia, which plays a crucial role in CNS homeostasis, the transmission of axon potentials, and the mediation of immune responses [36]. In vitro and in vivo studies have suggested that RTN4 is involved in the macrophage-associated inflammatory process [36]. A high expression of the NgR1/TROY/LINGO-1 receptor complex for RTN4 was revealed at the edge of chronic active demyelination lesions in multiple sclerosis (MS) patients in reactive microglia/macrophage and reactive astrocytes compared with controls. Moreover, more than 70% of microglia/macrophage cells were NgR1^+^ [38,43]. Additionally, recent studies revealed that overexpression of RTN4 may regulate the adhesion and polarization of macrophages and activate the secretion of M1 proinflammatory cytokines TNF-ɑ and IL-1β, whereas blocking of RTN4-A/B may weaken the M1-like phenotypes through the MAPK signaling pathway [44,45]. In light of the abovementioned facts, it seems crucial to explore the profound role of RTN4 protein in the regulation of macrophage-mediated inflammation. Considering that both proteins are markers of neurodegeneration and reflect other pathomechanisms underlying this disease, we calculated the NfL/RTN4 ratio to determine if these mutually complementary proteins in MS could be a valuable diagnostic tool. Our research showed that this coefficient enabled the differentiation between patients with MS and the control group. However, it did not appear to be a better biomarker than the NfL test alone.

In the present study, we also evaluated the diagnostic performance of CSF NfL, RTN4, and tau levels as clinical biomarkers enabling the discrimination of MS using ROC curves. The AUC values discriminating between controls and MS patients were higher for NfL than other neurodegenerative biomarkers (RTN4 and tau). Although further analysis revealed that this difference between NfL and RTN4 was insignificant, both biomarkers could be valuable in multiple sclerosis. Further studies comparing different neurodegenerative biomarkers are necessary, particularly in combination with imaging methods.

Our study has some limitations within which the findings need to be interpreted carefully. Firstly, in this study, we investigated a small population; however, we chose samples of patients with MS diagnosed in the same center and restored in the same facilities over similar periods. Additionally, collection procedures were standardized to avoid the influence of different conditions on the tested biomarker levels in our study cohort. Moreover, considering the promising results for NfL, our study will be continued, and other neurodegeneration biomarkers in the clinical practice of neurological disease will be explored and compared. Lastly, despite RTN4 reflecting major pathologies underlying MS (i.e., neurodegeneration and neuroinflammation), the combined analysis of NfL and RTN4 did not improve the accuracy of early diagnosis of MS patients. Therefore, additional empirical research is required to provide adequate answers regarding the early diagnosis of MS.

## 5. Conclusions

Neurodegeneration is a crucial problem underlying MS; thus, more studies are needed in this area. The best-known marker of neurodegeneration is NfL, although its specificity is limited. Therefore, further research should be carried out to find a panel of biomarkers enabling stratification of patients and individualized selection of effective therapy. Even though selected neurodegeneration indicators in physiological conditions share the same functions, their role in pathological conditions appears to differ. Our research suggests that CSF levels of NfL, RTN4, and tau alone could potentially be applied in the early diagnosis of MS. However, combined analysis of the tested proteins did not improve the accuracy of early diagnosis. Given the importance of this medical problem in MS, further research is still needed.

## Figures and Tables

**Table 1:** Participants’ demographic characteristics.

Variables	MS	Controls
Number	37	22
Sex		
Female	25	11
Male	12	11
Duration of disease, months	32	33
Oligoclonal bands, % positive (*n*)	76% (28)	0% (0/22)
Type 1 OCBs, % positive (*n*)	0%	22%
Type 2 OCBs, % positive (*n*)	82% (23)	0%
Type 3 OCBs, % positive (*n*)	18% (5)	0%
MRI demyelination changes, % positive (*n*)	78% (29)	0% (0/22)
T2 and FLAIR lesions, % positive (*n*)	45% (13)	0%
T2 or FLAIR lesions, % positive (*n*)	55% (16)	0%
Albumin serum (mg/dL)	4.36 ± 0.54	4.31 ± 0.65
IgG serum (g/L)	10.20 ± 2.03	9.27 ± 1.71
IgM serum (g/L)	1.46 ± 0.84	1.13 ± 0.46
IgA serum (g/L)	1.98 ± 0.66	2.24 ± 0.98
CRP (mg/dL)	3.5 ± 6.3	1.67 ± 1.32
WBC (10^3^/µl)	7.8 ± 2.05	6.9 ± 1.83
Glucose (mg/dL)	98 ± 23	99 ± 28
Cholesterol (mg/dL)	181 ± 46	204 ± 41
HDL (mg/dL)	47 ± 10	60 ± 21
LDL (mg/dL)	136 ± 48	140 ± 38
Comorbidities		
Hypertension, % positive	8%	23%
Spine disorders, % positive	16%	14%
Thyroid disorders, % positive	11%	9%
Type 2 diabetes, % positive	3%	5%
Eye diseases, % positive	11%	0%

Abbreviations: MS—multiple sclerosis, OCBs—oligoclonal bands, MRI—magnetic resonance imaging, FLAIR—fluid-attenuated inversion recovery, CRP—C-reactive protein, WBC—white blood cells.

**Table 2:** The cerebrospinal fluid concentrations of tested biomarkers.

CSF Data	MS	Controls	*p*-Value
	Mean ± SD	Mean ± SD	
Albumin (mg/dL)	203 ± 128	136 ± 91	0.041
Q_Alb_	5.85 ± 1.56	5.28 ± 2.20	0.3
IgG (mg/L)	5.22 ± 3.2	2.55 ± 0.85	<0.001
Q_IgG_	5.38 ± 3.14	2.77 ± 0.82	<0.001
IgM (mg/L)	1.70 ± 1.6	0.462 ± 0.32	0.002
Q_IgM_	1.18 ± 0.91	0.37 ± 0.19	<0.001
IgA (mg/L)	5.14 ± 4.5	3.52 ± 2.03	0.126
Q_IgA_	2.79 ± 2.82	1.57 ± 0.51	0.05
RTN4 (pg/mL)	96 ± 26	79 ± 25	0.012
NfL (pg/mL)	2059 ± 1845	778 ± 449	0.001
Tau (pg/mL)	211 ± 80	167 ± 82	0.05
NfL/RTN4	24 ± 26	11 ± 9	0.03
NfL/tau	10 ± 8	6 ± 7	0.091

Abbreviations: MS—multiple sclerosis, RTN4—reticulon 4, NfL—neurofilament light chain, Q_Alb_—albumin quotient, Q_IgG (A,M)_—immunoglobulins quotients.

**Table 3:** Diagnostic performance of various cerebrospinal fluid markers for multiple sclerosis.

Protein	Sensitivity (%)	Specificity (%)	PPV (%)	NPV (%)	AUC
NfL	78	77	85	68	0.836
RTN4	81	50	73	61	0.684
Tau	95	32	70	78	0.630

## Data Availability

The data presented in this study are available on request from the corresponding author. Key data are presented in the text.
